# Short-Listing the Program Choice for Perimetry in Neurological Conditions (PoPiN) Using Consensus Methods

**DOI:** 10.22599/bioj.143

**Published:** 2019-11-11

**Authors:** Lauren Hepworth, Fiona Rowe

**Affiliations:** 1 University of Liverpool, GB

**Keywords:** Perimetry, Visual field loss, Idiopathic intracranial hypertension, Chiasmal compression, Stroke, Optic neuropathy

## Abstract

**Background:**

Neurological conditions frequently cause visual field loss, commonly resulting in perimetry requests for suspected or known conditions. Currently there are no national guidelines for perimetry in neurological conditions. A wide choice of perimetry programs exists. An inappropriate program choice could fail to detect visual field loss. Two phases in this study determined preference of perimetry programs for detection of visual field loss in four common neurological conditions (idiopathic intracranial hypertension (IIH), optic neuropathies, chiasmal compression and stroke), to aid the design of research and clinical practice guidelines.

**Methods:**

A survey consisted of 47 perimetry programs. Orthoptists and neuro-ophthalmologists were asked which perimetry programs they considered important for use in the four neurological conditions. These programs were short-listed for discussion in a consensus meeting. A nominal group technique was used for the consensus meeting to reach consensus on the three most favoured perimetry programs appropriate for the four conditions.

**Results:**

Twenty-six participants completed the survey (51% return rate). Nine programs were found to be not applicable to any of the conditions. The short-lists for the conditions varied between six and ten perimetry programs. Seven participants discussed the survey results at a consensus meeting to agree the three most favoured perimetry programs for IIH, optic neuropathy and chiasmal compression (manual/semi manual kinetic, static 30–2 and full-field 120) and for stroke (manual/semi manual kinetic, static 30–2 and monocular Esterman).

**Conclusion:**

A wide range of perimetry programmes were explored thoroughly through survey and consensus methods in order to determine clinician preference for their use in neuro-ophthalmic practice. The three most favoured perimetry programs for the four conditions was established.

## Introduction

The common neurological conditions which present to hospital eye services include idiopathic intracranial hypertension (IIH), optic neuropathies, chiasmal compression and stroke (Johnson & Keltner 1983). Perimetry has three important functions in neuro-ophthalmology for these conditions; diagnosis, monitoring and assessment of visual function (Kedar et al. 2011).

The monitoring of visual fields in IIH is crucial as onset can be insidious, asymptomatic and occur at any stage (Corbett et al. 1982; Pane et al. 2007). Visual field loss is an important component in the diagnosis of optic neuropathy (Hayreh & Zimmerman 2005). There are many aetiologies of optic neuropathy, the most common including optic neuritis and anterior ischaemic optic neuropathy (AION) (Pane et al. 2007). Chiasmal compression commonly presents with visual field loss. The detection of peripheral visual field loss is crucial in allowing early diagnosis and prompt intervention (Rowe et al. 2015). Approximately one third of stroke survivors have been reported to have visual field loss (Rowe et al. 2019). Current UK national guidelines advised the presence of visual field loss should be tested for in every patient after stroke (Intercollegiate Stroke Working Party 2016). Recovery of stroke-related visual field defects can be monitored with repeated perimetry (Jones & Shinton 2006).

Perimetry in patients with acute and chronic ocular and/or neurological diseases is an important clinical tool and a ‘corner-stone’ assessment within ophthalmology. Recommendations currently exist for visual field assessment in glaucoma but not for neurological conditions (National Institute for Health and Clinical Excellence 2009). The recommendation for the 24–2 program to be used as the reference standard in glaucoma has streamlined clinical practice, providing clinical results that clinicians globally accept and recognise. To afford the same benefits to other commonly occurring conditions there is a pressing need to have reference standards for the visual field programs to use for neurological conditions.

The diagnosis of neurological pathology can be delayed by a missed or delayed identification of visual field loss, when the area tested by the perimetry program does not overlap with the visual field defect, which can have life changing consequences. In view of this, diagnostic accuracy of visual field assessment is crucial.

The first stage of this programme of research on Perimetry in Neurological Conditions (PoPiN) was a systematic review which identified a wide range of perimetry programs used and current lack of standardisation in perimetry assessment for four commonly occurring neurological conditions (Hepworth & Rowe 2018). It found the most commonly used static perimetry programs to be the 30–2 and 24–2 on the Humphrey II-i, both of which assess the central visual field. By only assessing the central visual field, the detection of any defects beyond these boundaries is limited, reducing diagnostic accuracy in some conditions where the peripheral visual field is affected first (Khoury et al. 1999; Rowe et al. 2015). The Goldmann perimeter was reported as the second most commonly used perimeter for manual kinetic perimetry. This systematic review also found that the patterns of visual field defects varied greatly across the four conditions (Hepworth & Rowe 2018). The 24–2 program has been found to be used extensively in neurological conditions (Hepworth & Rowe 2018). This is despite a lack of supporting evidence for the diagnostic accuracy of this program for neurological conditions, and especially concerning for conditions where the peripheral visual field is affected first and the central visual field is only affected after significant progression.

The aim of this study is to develop a consensus on the three most favoured visual field programs, considered most suited for visual field loss detection, for four commonly occurring neurological conditions (chiasmal compression, IIH, stroke and optic neuropathy).

## Methods

This study comprises two phases: (1) a survey to identify a short list of visual field programs for the four neurological conditions and (2) consensus meeting to agree the three most favoured visual field programs for each of the four neurological conditions. Ethical permission for this study was granted by the University of Liverpool Research Ethics Commitee (IPHS-2643).

### Phase 1: Survey

Clinicians, i.e. orthoptists and neuro-ophthalmologists, with knowledge of visual impairment following one or more of the neurological conditions were targeted. An advertisement outlining the survey was used to identify participants through professional networks. Participants emailed the research team to express interest in participating.

The survey remained open for four weeks and was delivered via the Survey Monkey™ platform. The opening page of the survey included information about the purpose and content of the survey. Implied informed consent was deemed to have been obtained from those who completed the survey.

Forty-seven perimetry programs were included in the survey, outlined in Table 1, identified from a systematic review and manufacturer perimeter manuals (Hepworth & Rowe 2018). Participants were asked to select the perimetry programs important for use in each of the four neurological conditions, with the stipulation these did not have to be the programs they currently use. An option of not applicable to any of the four conditions was also given.

**Table 1:** List of perimetry programs included in survey.

			Program name	

**STATIC**	CENTRAL	10°	10–2 (H, O)	
			Macula (O)	
			M-program (O)	
			N-fovea (O)	
		
		20°	24–2 (H, O)	
			Central Armaly screening (H)	
			N-blind spot (O)	
		
		30°	30–2 (H, O)	
			G-program (O)	
			Central 40 (H)	
			Central 64 (H)	
			Central 76 (H)	
			Central 80 (H)	
			Low vision central (O)	

	PERIPHERAL	50°	BG (Blindengutachten) pattern (O)	
			D-pattern (O)	
			Full field Armaly screening (H)	
			Nasal step (H)	*

		60°	60–4 (H, O)	
			Full field 81 (H)	
			Full field 120 (H)	
			Full field 246 (H)	
			Peripheral 60 (H)	
			Peripheral 68 (O)	

		70°	07 pattern (O)	
			N-full field (O)	

			FG (Führerscheingutachten) pattern (O)	

		80°	Esterman Binocular (H, O)	
			Esterman Monocular (H, O)	
			Blepharoptosis pattern (O)	*

		90°	Full field 135 (H)	
			Low vision peripheral (O)	

**KINETIC**	C	30°	Octopus Blindspot (O)	

	PERIPHERAL	90°	Manual Kinetic (G, H, O)	
			Examiner-led semi manual (O)	
			Octopus General (O)	
			Octopus Altitudinal (O)	*
			Octopus Driving/Screening (O)	
			Octopus Hemianopia (O)	*
			Octopus Pituitary (O)	*
			Octopus Quadrantanopia (O)	*


(G) = Goldman (H) = Humphrey, (O) = Octopus, * = condition specific.

### Phase 2: Consensus Meeting

A consensus approach was sought using a focus group in which information was collected through a semi-structured group interview process. A norminal group technique consisting of the following steps was used for each of the four conditions (Cantrill et al. 1996):

1. Verbal and written presentation of the data set from the survey and systematic review,
2. Generation of ideas and opinions in silence,
3. Feedback from each participant in turn to the group, listed by the facilitator on a flip-chart for reference during the discussion,
4. Group discussion regarding the feedback,
5. Voting and decision agreement regarding the three most favoured perimetry programs.

The consensus definition used for the session when voting was, an acceptable resolution, one that can be supported, even if not the ‘favourite’ of each individual.

Participants, patients and clinicians with knowledge of visual impairment following one or more of the neurological conditions were targeted. An advertisement outlining the meeting was disseminated by professional and patient groups in order to identify participants. Participants emailed the research team if expressing interest in participating. Written informed consent was sought prior to the start of the meeting.

## Results

**Figure 1:**
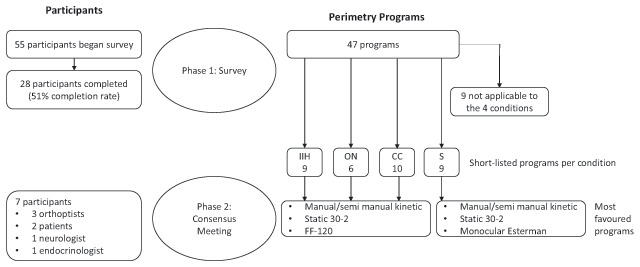
Flow chart of participants and perimetry programs through the study. IIH = intracranial hypertension, ON = optic neuropathy, CC = chiasmal compression, S = stroke.

An overview of the perimetry programs and participants involved in the study is outlined in Figure 1.

### Phase 1: Survey

Fifty-five participants started the survey, with 28 participants completing it (51% completion rate). The professions of those that completed the survey were orthoptists (n=27) and one neuro-ophthalmologist. The majority (71.4%) had more than ten years clinical experience and all had more than one year of clinical experience. Of the 27 participants who did not start the main body of the survey, 25 were orthoptist, one was an ophthalmic technician and one unknown, of which the majority also had more than ten years clinical experience (74.1%). The groups who completed or did not complete the survey had similar professional backgrounds and level of experience.

Nine (19.1%) of the 47 programs received not applicable responses without being selected as important for any of the four conditions by any participant: Blepharoptosis pattern, Full Field Armaly Screening, Full Field-135, Full Field-246, M-Program, Nasal Step, Peripheral 60, Peripheral 68, 07 Pattern.

The programs short-listed for each of the four neurological conditions are summarised in Table 2.

**Table 2:** List of perimetry programs short-listed for each condition by the survey.

Idiopathic intracranial hypertension	Optic neuropathy	Chiasmal compression	Stroke

Manual kinetic	Manual kinetic	Manual kinetic	Manual kinetic
30–2	30–2	30–2	30–2
24–2		24–2	
Binocular Esterman	Binocular Esterman	Binocular Esterman	Binocular Esterman
Examiner-led Semi-kinetic Octopus 900		Examiner-led Semi-kinetic Octopus 900	Examiner-led Semi-kinetic Octopus 900
Monocular Esterman		Monocular Esterman	Monocular Esterman
Full-Field 120	Full-Field 120	Full-Field 120	Full-Field 120
Octopus Blind-spot			
Octopus Driving/Screening		Octopus Driving/Screening	Octopus Driving/Screening
	Macula Octopus		
		Octopus Hemianopia	Octopus Hemianopia
		Octopus Pituitary	
			Octopus quadrantanopia

#### Idiopathic intracranial hypertension

The survey produced a short-list of nine perimetry programs: manual kinetic, 30–2, 24–2, Binocular Esterman, Examiner-led Semi-kinetic Octopus 900, Monocular Esterman, Full-Field 120, Octopus Blind-spot, Octopus Driving/Screening.

#### Optic neuropathy

The survey produced a short-list of six perimetry programs: manual kinetic, 24–2, 30–2, Binocular Esterman, Full-Field 120, Macula Octopus.

#### Chiasmal compression

The survey produced a short-list of 10 perimetry programs: manual kinetic, Binocular Esterman, Monocular Esterman, Examiner-led Semi-kinetic Octopus, Full-Field 120, Octopus Pituitary, 30–2, Octopus Driving/Screening, Octopus Hemianopia, 24–2.

#### Stroke

The survey produced a short-list of nine perimetry programs: Binocular Esterman, manual kinetic, Monocular Esterman, Examiner-led Semi-kinetic Octopus, Full-Field 120, Octopus Driving/Screening, Octopus Hemianopia, Octopus quadrantanopia, 30–2.

### Phase 2: Consensus Meeting

The consensus meeting was a half day event held in Liverpool, UK with seven participants comprising three orthoptists, two patients with experience of having perimetry (one with a pituitary tumour and one stroke survivor), one neurologist and one endocrinologist.

Prior to discussing the four separate conditions there was a discussion of the practicalities relating to visual field assessment. Patient preference was for quicker assessments, and preferably less than 10 minutes in duration. Further discussion addressed test durations in relation to the impact on reliability, and consideration for the overall physical and cognitive condition of patients with neurological conditions for cooperation with testing.

The group also considered that whilst it is important to provide the best possible perimetry results, further information can be gained from imaging, other clinical tests and ophthalmoscopy. Therefore visual fields are not taken as the sole source of information in patient diagnosis and management decisions.

For all four neurological conditions, consensus was obtained regarding manual kinetic perimetry excluding the use of Humphrey perimeter due to the ceiling effects for the superior visual field (Rowe et al. 2019), and specifying if kinetic perimetry is done using the Octopus 900, this should be completed using the standardised speed settings of the semi-kinetic mode.

#### Idiopathic intracranial hypertension

From the nine perimetry programs short-listed by the survey, consensus was obtained to completely exclude five programs detailed in Table 3(a); 24–2, Binocular Esterman, Monocular Esterman, Octopus Blind-spot and Octopus Driving/Screening.

**Table 3:** Reason for omitting test from short list in consensus meeting.

Program name	Included	Excluded	Reasons/comments

**(a) Idiopathic intracranial hypertension**			

Manual Kinetic (G, H, O)	•	•	Humphrey kinetic due to ceiling effects at 50° [14]. If Octopus use with standard speed settings (semi-manual)
30–2 (H, O)	•		
24–2 (H, O)		•	Blind spot not fully captured
Esterman Binocular (H, O)		•	Not appropriate for monitoring due to lack of threshold sensitivity
Examiner-led semi manual (O)	•		
Esterman Monocular (H, O)		•	Not appropriate for monitoring due to lack of threshold sensitivity
Full field 120 (H)	•		
Octopus Blindspot (O)		•	Not available on all Octopus perimeters
Octopus Driving/Screening (O)		•	Not available on all Octopus perimeters
**(b) Optic neuropathy**			

Manual Kinetic (G, H, O)	•	•	Humphrey kinetic due to ceiling effects at 50° [14]. If Octopus use with standard speed settings (semi-manual).
24–2 (H, O)		•	Blind spot not fully captured
30–2 (H, O)	•		
Esterman Binocular (H, O)		•	Not appropriate for monitoring due to lack of threshold sensitivity
Full field 120 (H)	•		
Macula (O)		•	Not a screening test. Defer to RCOphth guidelines if patient on hydroxycholrequine (Royal College of Ophthalmologists, 2018)
**(c) Chiasmal compression**			

Manual Kinetic (G, H, O)	•	•	Humphrey kinetic due to ceiling effects at 50° [14]. If Octopus use with standard speed settings (semi-manual)
Esterman Binocular (H, O)		•	Not appropriate for monitoring due to lack of threshold sensitivity
Esterman Monocular (H, O)		•	Not appropriate for monitoring due to lack of threshold sensitivity
Examiner-led semi manual (O)	•		
Full field 120 (H)	•		
Octopus Pituitary (O)		•	Not available on all Octopus perimeters
30–2 (H, O)	•		
Rowe – Driving/Screening (O)		•	Not available on all Octopus perimeters
Octopus Hemianopia (O)		•	Not available on all Octopus perimeters
24–2 (H, O)		•	Blind spot not fully captured
**(d) Stroke**			

Esterman Binocular (H, O)		•	
Manual Kinetic (G, H, O)	•	•	Humphrey kinetic due to ceiling effects at 50° [14]. If Octopus use with standard speed settings (semi-manual)
Esterman Monocular (H, O)	•		
Examiner-led semi manual (O)	•		
Full field 120 (H)		•	Distribution of stimuli locations too focused in the nasal field
Octopus Driving/Screening (O)		•	Not available on all Octopus perimeters
Octopus Hemianopia (O)		•	Not available on all Octopus perimeters
Octopus Quadrantanopia (O)		•	Not available on all Octopus perimeters
30–2 (H, O)	•		

The three most favoured perimetry programs agreed for idiopathic intracranial hypertension at the consensus meeting were manual/semi manual kinetic, static 30–2 and FF-120.

#### Optic neuropathy

From the six perimetry programs short-listed by the survey, consensus was obtained to completely exclude three programs detailed in Table 3(b); 24–2, Binocular Esterman and Macula. The three most favoured perimetry programs agreed for optic neuropathy at the consensus meeting were manual/semi manual kinetic, static 30–2 and FF-120.

#### Chiasmal compression

From the ten perimetry programs short-listed by the survey, consensus was obtained to completely exclude six programs detailed in Table 3(c); Binocular Esterman, Monocular Esterman, Octopus Pituitary, Octopus Driving/Screening, Octopus Hemianopia and 24–2. The three most favoured perimetry programs agreed for chiasmal compression at the consensus meeting were manual/semi manual kinetic, static 30–2 and FF-120.

#### Stroke

From the nine perimetry programs short-listed by the survey, consensus was obtained to completely exclude five programs detailed in Table 3(d); Binocular Esterman, Full-Field 120, Octopus Driving/Screening, Octopus Hemianopia, Octopus quadrantanopia.

The three most favoured perimetry programs agreed for stroke at the consensus meeting were manual/semi manual kinetic, static 30–2 and monocular Esterman.

## Discussion

We report the results of a survey and consensus process to determine the three most favoured perimetry programs deemed best suited for the visual field assessment in four neurological conditions (chiasmal compression, IIH, optic neuropathy and stroke) that commonly present to eye clinics. This is the second stage of a programme of research (PoPiN) for this purpose. The first stage was a systematic review to identify patterns of visual field loss and types of perimetry programs used to assess visual fields in four common neurological conditions. The systematic review identified 20 programs which populated this survey and consensus process, with an additional 27 obtained from manufacturer perimeter manuals (Hepworth & Rowe 2018). The survey fullfied its role of narrowing perimetry programs across the four target conditions in advance of subsequent discussion of these in a consensus meeting.

Following the consensus meeting the same three programs were selected as most favoured for use in three of the neurological conditions considered in this study; chiasmal compression, IIH and optic neuropathy. These included manual/semi-manual kinetic perimetry, static 30–2 and FF-120 programmes. For stroke two of the three most favoured programs were consistent with the other three conditions; manual/semi manual kinetic, static 30–2. The FF-120 program selected in the other conditions was replaced by the monocular Esterman program for stroke; the reason being the distribution of stimuli locations are clustered closely in the nasal visual field but more sparsely located in the temporal visual field in the FF-120. A systematic review reported both the static 30–2 program and manual kinetic perimetry were commonly used in studies across all four conditions (Hepworth & Rowe 2018).

Delphi techniques and nominal group techniques are commonly used consensus methodologies (McMillian et al. 2016). This study did not use Delphi technique to inform the nominal group meeting. A Delphi survey would ask participants to express their opinion on the importance of each of the perimetry programs through a ranking process. This would have provided more detailed information on the degree of importance placed on each program from a wider population as a starting pointing for the consensus meeting. However. this study opted not to use the Delphi technique because of the low completion rates with this technique seen in eye research (Al Jabri et al. 2019; Rowe et al. 2019). Instead we favoured using results from a systematic review to populate a shorter survey to maximise the return rate. In the absence of ranking information each perimetry program selected as important for use from the survey was considered equally in the nominal group meeting.

This study has taken a step closer in providing information to develop clinical guidelines on which perimetry programs are optimal for four common neurological conditions which routinely present to eye clinic. It is important to target the correct choice of perimetry programme for these common neurological conditions. IIH has visual field impairment predominantly due to papilloedema and its effect on the optic disc and retinal nerve fibre layers. Thus visual field assessment should seek to capture the extent of blind spot enlargement and related retinal nerve fibre layers such as arcuate defects and paracentral scotomas (Wall & George 1991; Cello et al. 2016). Chiasmal compression most frequently causes heteronymous visual field loss such as bitemporal hemianopia which may affect the peripheral temporal visual field prior to involvement of the central visual field (Rowe et al. 1995; Rowe et al. 2015). Optic neuropathies can present with a variety of visual field defects ranging from central scotoma to arcuate defects and constricted visual fields. Perimetry programmes should aim to capture this wide range of visual field loss (Keltner et al. 2003; Hayreh & Zimmerman 2005; Keltner et al. 2010). Stroke may affect any part of the visual pathway from the eye (in ocular stroke with altitudinal visual field defects) through to the occipital cortex with typical retrochiasmal visual field defects of homonymous hemianopia, quadrantanopia and scotomas; peripheral and/or central visual fields may be involved so quire appropriate perimetry programme choice (Rowe et al. 2013; Hanna et al. 2017).

The use of consistent methods of visual field assessment brought about by glaucoma recommendations have streamlined clinical practice (National Institute for for Health and Clinical Excellence 2009). There are also benefits for such guidelines for research, in allowing future meta-analysis for treatment options of these conditions. Our systematic review revealed a wide range of perimetry programs are being used across studies of the four neurological conditions (Hepworth & Rowe 2018). Any future recommendations require a strong evidence base, and further research is needed to decide which perimetry programs have the best diagnostic accuracy and are most appropriate for early detection in each condition. This study has narrowed the programs to be included in such clinical research.

## Conclusion

Consensus has been obtained on the three most favoured perimetry programs for comparison of use in four common neurological conditions to aid the refinement in choice of testing for visual field loss. The same three most favoured programs were selected for chiasmal compression, IIH and optic neuropathy: manual/semi manual kinetic, static 30–2 and monocular Esterman. For stroke the three most favoured programs were manual/semi manual kinetic, static 30–2 and FF-120. Further research is now required to compare the diagnostic accuracy of these three visual field assessments for each of the target conditions in order to improve accuracy of testing choice, and to inform practice guidelines and clinical decision making for the assessment of these conditions.

## References

[1] Al Jabri, S, Kirkham, JJ and Rowe, FJ. 2019 Development of a core outcome set for amblyopia, strabismus and ocular motility disorders: A review to identify outcome measures. BMC Ophthalmology, 19 DOI: 10.1186/s12886-019-1055-8 PMC6368710 30736755

[2] Cantrill, JA, Sibbald, B and Buetow, S. 1996 The Delphi and nominal group techniques in health services research. The International Journal of Pharmacy Practice, 4: 67–74. DOI: 10.1111/j.2042-7174.1996.tb00844.x

[3] Cello, KE, Keltner, JL, Johnson, CA, Wall, M and Nordic Idiopathic Intracranial Hypertension Study Group. 2016 Factors affecting visual field outcomes in the Idiopathic Intracranial Hypertension Treatment Trial. Journal of Neuro-Ophthalmology, 36: 6–12. DOI: 10.1097/WNO.0000000000000327 26618282

[4] Corbett, JJ, Savino, PJ, Thompson, S, Kansu, T, Schatz, NJ, Orr, LS and Hopson, D. 1982 Visual loss in pseudotumour cerebri. Archives of Neurology, 39: 461–474. 7103794 10.1001/archneur.1982.00510200003001

[5] Hanna, KL, Hepworth, LR and Rowe, FJ. 2017 Screening methods for post-stroke visual impairment: A systematic review. Disability and Rehabilitation, 39: 2531–2543. DOI: 10.1080/09638288.2016.1231846 27669628

[6] Hayreh, SS and Zimmerman, B. 2005 Visual field abnormalities in nonarteritic anterior ischemic optic neuropathy their pattern and prevalence at initial examination. Archives of Ophthalmology, 123: 1554–1562. DOI: 10.1001/archopht.123.11.1554 16286618

[7] Hepworth, LR and Rowe, FJ. 2018 Programme choice for perimetry in neurological conditions (PoPiN): A systematic review of perimetry options and patterns of visual field loss. BMC Ophthalmology, 18 DOI: 10.1186/s12886-018-0912-1 PMC6131852 30200926

[8] Intercollegiate Stroke Working Party. 2016 National clinical guideline for stroke. London: Royal College of Physicians.

[9] Johnson, CA and Keltner, JL. 1983 Incidence of visual field loss in 20,000 eyes and its relationship to driving performance. Archives of Ophthalmology, 101: 371–375. DOI: 10.1001/archopht.1983.01040010371002 6830485

[10] Jones, SA and Shinton, RA. 2006 Improving outcome in stroke patients with visual problems. Age and Ageing, 35: 560–565. DOI: 10.1093/ageing/afl074 16820528

[11] Kedar, S, Ghate, D and Corbett, JJ. 2011 Visual fields in neuro-ophthalmology. Indian Journal of Ophthalmology, 59: 103–109. DOI: 10.4103/0301-4738.77013 21350279 PMC3116538

[12] Keltner, JL, Johnson, CA, Cello, KE, Dontchev, M, Gal, RL, Beck, RW and Optic Neuritis Study Group. 2010 Visual field profile of optic neuritis: A final follow-up report from the optic neuritis treatment trial from baseline through 15 years. Archives of Ophthalmology, 128: 330–337. DOI: 10.1001/archophthalmol.2010.16 20212204 PMC4107874

[13] Keltner, JL, Johnson, CA, Cello, KE, Edwards, MA, Bandermann, SE, Kass, MA, Gordon, MO and OHTS Group. 2003 Classifcation of visual field abnormalities in the Ocular Hypertension Treatment Study. Archives of Ophthalmology, 121: 643–650. DOI: 10.1001/archopht.121.5.643 12742841

[14] Khoury, JM, Donahue, SP, Lavin, PJM and Tsai, JC. 1999 Comparison of 24–2 and 30–2 perimetry in glaucomatous and non-glaucomatous optic neuropathies. Journal of Neuro-Ophthalmology, 19: 100–108. DOI: 10.1097/00041327-199906000-00004 10380130

[15] McMillian, SS, King, M and Tully, MP. 2016 How to use the nominal group and Delphi techniques. International Journal of Clinical Pharmacy, 38: 655–662. DOI: 10.1007/s11096-016-0257-x 26846316 PMC4909789

[16] National Institute for for Health and Clinical Excellence. 2009 Glaucoma: Diagnosis and management of chronic open angle glaucoma and ocular hypertension. London: National Collaborating Centre for Acute Care at The Royal College of Surgeons of England. 21938863

[17] Pane, A, Burdon, M and Miller, NR. 2007 The neuro-ophthalmology survival guide. Edinburgh, UK: Mosby Elsevier.

[18] Rowe, F, Thompson, C and Webster, A. 1995 Incidence of bitemporal hemianopic visual field defects in pituitary tumours, 279–283. Kyoto, Japan: International Orthoptic Congress.

[19] Rowe, FJ, Chenye, CP, Garcia-Fiñana, M, Noonan, C, Howard, C, Smith, J and Adeoye, J. 2015 Detection of visual field loss in pituatary disease: Peripheral kinetic versus central static. Neuro-Ophthalmology, 39: 116–124. DOI: 10.3109/01658107.2014.990985 27928344 PMC5123138

[20] Rowe, FJ, Hepworth, LR, Hanna, KL, Mistry, M and Noonan, CP. 2019 Accuracy of kinetic perimetry assessment with the Humphrey 850; An exploratory comparative study. Eye. DOI: 10.1038/s41433-019-0520-1 PMC7002568 31332292

[21] Rowe, FJ, Hepworth, LR, Howard, C, Hanna, KL, Cheyne, CP and Currie, J. 2019 High incidence and prevalence of visual problems after acute stroke: An epidemiology study with implications for service delivery. PLoS One, 14 DOI: 10.1371/journal.pone.0213035 PMC6402759 30840662

[22] Rowe, FJ, Wright, D, Brand, D, Jackson, C, Harrison, S, Maan, T, Scott, C, Vogwell, L, Peel, S, Akerman, N, Dodridge, C, Howard, C, Shipman, T, Sperring, U, MacDiarmid, S and Freeman, C. 2013 A prospective profile of visual field loss following stroke: Prevalence, type, rehabilitation and outcome. BioMed Research International, 2013 DOI: 10.1155/2013/719096 PMC3782154 24089687

[23] Royal College of Ophthalmologists. 2018 Hydroxychloroquine and chloroquine retinopathy: Recommendations on screening. London: Royal College of Ophthalmologists. 10.1038/s41433-018-0136-x PMC6043500 29887605

[24] Wall, M and George, D. 1991 Idiopathic intracranial hypertension. A prospective study of 50 patients. Brain, 114: 155–180. 1998880

